# Supplementary material for: Investigation of Metal-Organic Framework-5 (MOF-5) as an Antitumor Drug Oridonin Sustained Release Carrier
Source: Molecules. 2019 Sep 16;24(18):3369. doi: 10.3390/molecules24183369 (PMC6767262; doi:10.3390/molecules24183369)
Supplement: Supplementary file 1 [file molecules-24-03369-s001.pdf]

Investigation of Metal-Organic Framework-5 (MOF-5) as Antitumor Drug Oridonin Sustained release Carrier

Gongsen Chen[1], Juyuan Luo[1], Mengru Cai[1], Liuying Qin[1], Yibo Wang[1], Lili Gao[1], Pingqing Huang[1], Yingchao Yu[1], Yangming Ding[1], Xiaoxv Dong [1], Xingbin Yin[1**] and Jian Ni[1,2*]

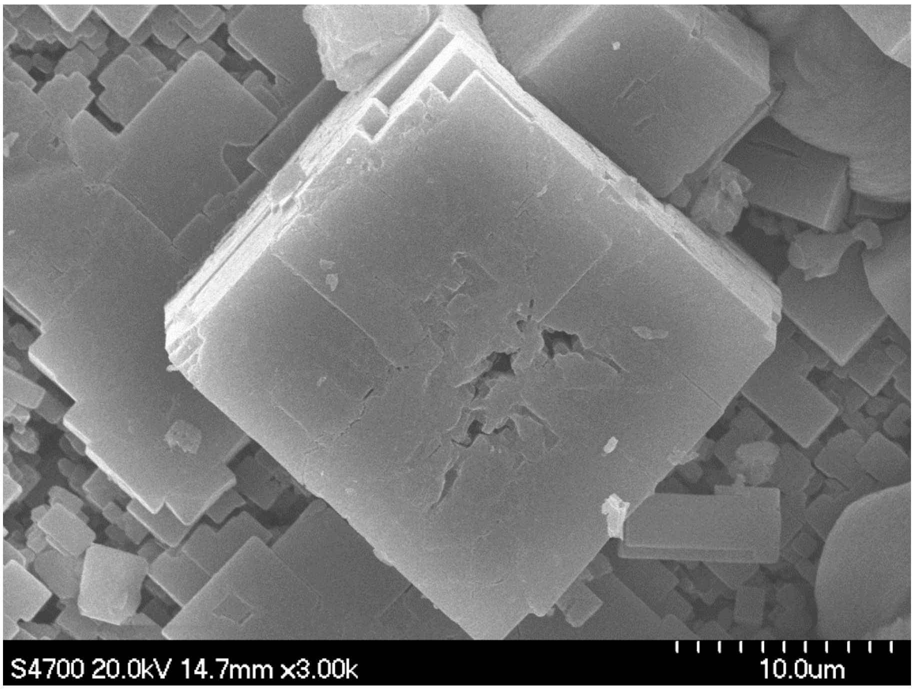

1 MOF-5-S

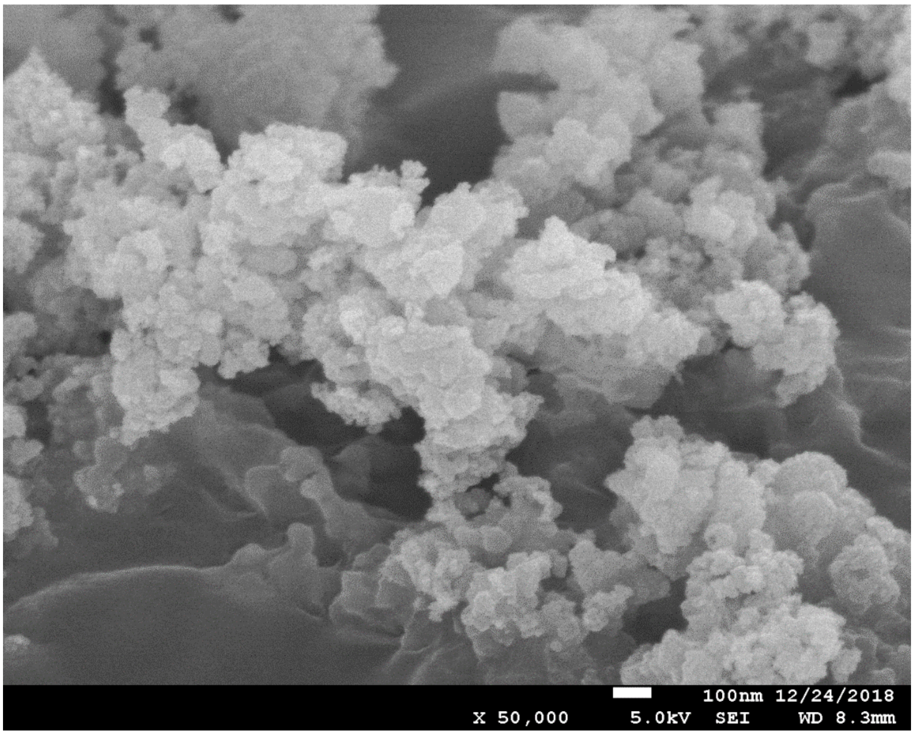

2 MOF-5-D
